# Indices of Loading and Propulsive Ability in the Gait of Patients With Chronic Stroke With Equinus Foot Deviation: A Correlation Study

**DOI:** 10.3389/fnhum.2021.771392

**Published:** 2022-01-14

**Authors:** Davide Mazzoli, Giacomo Basini, Paolo Prati, Martina Galletti, Francesca Mascioli, Chiara Rambelli, Paolo Zerbinati, Isabella Campanini, Andrea Merlo

**Affiliations:** ^1^Gait and Motion Analysis Laboratory, Sol et Salus Hospital, Rimini, Italy; ^2^Section of Rehabilitation, Department of Neuroscience, University of Padova, Padova, Italy; ^3^Neuro-Orthopedic Unit, Sol et Salus Hospital, Rimini, Italy; ^4^LAM-Motion Analysis Laboratory, Department of Neuromotor and Rehabilitation, Azienda USL-IRCCS di Reggio Emilia, S. Sebastiano Hospital, Correggio, Italy

**Keywords:** gait, stroke, equinus foot deviation, ground reaction force, rehabilitation, physiotherapy

## Abstract

In literature, indices of overall walking ability that are based on ground reaction forces have been proposed because of their ease of administration with patients. In this study, we analyzed the correlation between the indices of dynamic loading and propulsion ability of 40 chronic hemiparetic post-stroke patients with equinus foot deviation and a set of clinical assessments of ankle joint deviations and walking ability. Ankle passive and active range of motion (ROM) and triceps surae spasticity were considered, along with walking speed and three complementary scales of walking ability focusing respectively on the need for assistance on functional mobility, including balance and transfers, and the limitation in social participation. The correlation between the ground reaction force-based indices and both clinical and functional variables was carried out using the non-parametric Spearman correlation coefficient. Both indices were correlated to 8 of the 10 investigated variables, thus supporting their use. In particular, the dynamic propulsive ability was correlated with all functional scales (rho = 0.5, *p* < 0.01), and has the advantage of being a continuous variable. Among clinical assessments, limited ankle ROM affected walking ability the most, while spasticity did not. Since the acquisition of ground reaction forces does not require any patient prepping, the derived indices can be used during the rehabilitation period to quickly detect small improvements that, over time, might lead to the broad changes detectable by clinical scales, as well as to immediately highlight the lack of these improvements, thus suggesting adjustments to the ongoing rehabilitation approach.

## Introduction

Stroke is a major cause of disability worldwide. The number of subjects facing a post-stroke condition continues to rise due to the increase and aging of the global population and to the decrease in mortality associated with acute vascular events (Patrick and Keenan, 2007; Katan and Luft, 2018) People who survive this acute event can develop pathological gait patterns, which eventually lead to a decreased deambulatory performance in terms of walking speed, energy expenditure, safety, and pain (Kesar et al., 2011). An impaired walking ability strongly contributes to the reduction of the independence of a patient and leads to a decrease in social participation (Perry et al., 1995).

Pathological walking patterns originate from a combination of factors, including the loss in muscle strength, inappropriate muscle activation, altered exteroceptive or proprioceptive perception, and the development of joint deformities. Focusing on the lower limb, the equinus and equinovarus foot deviations (EVD, EVFD) are the most common lower limb deformity in stroke survivors (Giannotti et al., 2018) along with the limitation in knee flexion referred to as stiff knee gait (SKG) (Merlo and Campanini, 2019).

In clinical practice, the assessment of pathophysiological conditions underlying joint deviations and altered motor function typically rely on a clinical evaluation at the bedside [e.g., range of motion (ROM), force, and spasticity], on visual assessment when walking and on the use of a set of walking-related clinical scales and functional tests that are used to assess the walking ability of a patient in different settings (e.g., indoor/outdoor) with or without assistance.

When designing the rehabilitation plan for a patient, the instrumental assessment of gait can be used to answer specific questions that arise from the clinical assessment, and which cannot be answered by the clinical investigation itself (Baude et al., 2019; Campanini et al., 2020). Furthermore, in patients with stroke, the presence or absence of specific muscle activation patterns (e.g., spasticity) at the bedside evaluation, does not necessarily predict the presence or absence of the same patterns while standing and walking (Gracies, 2005; Baude et al., 2019; Merlo and Campanini, 2019; Campanini et al., 2020).

While being used in the decision-making process, a comprehensive clinical and instrumental assessment cannot be performed to monitor the patient evolution throughout the whole treatment period, due to its time requirements and costs. In literature, instrumental indices computed from ground reaction force (GRF) data, such as the dynamic loading ability (DLA) and the peak or mean dynamic propulsive ability (DPA), have been suggested to overcome this issue (Morita and Yamamoto, 1995; Turns et al., 2007; Campanini and Merlo, 2009; Raja et al., 2012; Roelker et al., 2019). Since the GRF acquisition does not require any patient prepping (e.g., markers, electrodes), this instrumental assessment can be completed in a few minutes and could be reasonably used during the rehabilitation period to complement the information provided by clinical assessments and walking speed. Moreover, the indices can be obtained even with patients wearing orthoses or using walking aids thus providing a quick tool to compare the effect of different orthoses (Bowden et al., 2006; Campanini and Merlo, 2009).

Because of the potential usefulness of DLA and DPA in the assessment of the recovery of walking ability in patients and developed EVFD, in this study, we analyzed the correlation between the clinical assessment of EVFD and walking ability and the instrumental assessment of walking provided by the indices.

## Materials and Methods

### Study Design and Setting

In this correlation study, we retrospectively analyzed data from patients with stroke with chronic hemiparesis who were referred to our laboratory for the detailed identification of the causes of their joint deviations to support treatment selection.

### Participants

Patients were included in this study based upon the following criteria: (1) ischemic or hemorrhagic stroke; (2) right or left hemiparesis; (3) time elapsed since the stroke >6 months; (4) ability to walk barefoot and without assistance at a self-selected speed for 10 meters; (5) available clinical and GRF data; (6) available signed informed consent of data collection and usage for clinical research purposes. The exclusion criteria were: (1) any other neurological or orthopedic disease that could limit their ability to walk; (2) any previous lower limb surgical intervention; (3) any focal inhibition on the lower limb muscles during the 6 months prior to evaluation.

This study was approved by the Local Ethics Committee (CEROM, protocol 5953/2017).

### Clinical Assessment

Based on the clinical assessment we extracted the following variables from the records of the patients: age, sex, affected side, the time elapsed from stroke and its etiology, the maximum passive ankle dorsiflexion measured with the knee both extended and flexed at 90°, the plantar flexor muscles spasticity assessed by the Modified Tardieu Scale (MTS) and with the knee in both positions, and the strength of plantar and flexor muscles assessed using the Medical Research Council (MRC) scale.

Walking ability was assessed by three complementary scales: the Functional Ambulation Category (FAC), the Rivermead Mobility Index (RMI), and the Walking Handicap Scale (WHS). FAC is a quick visual measurement of walking ability (Mehrholz et al., 2007). It ranges between 0 (non-walking patient) and 5 (independent ambulator or any surface), with intermediate scores indicating the amount of assistance needed to walk. RMI ranges between 0 and 15 and covers a clinically relevant range of activities from the ability to turn in bed, to sit, to stand, walking inside and outside with and without help, up to running (Collen et al., 1991). For WHS, a range of 0–6 is used to distinguish between housebound patients and walkers with community potential (Perry et al., 1995). Direct measurement of walking speed was also recorded.

### Instrumental Assessment

Force plate data, which are the focus of this study, were acquired by means of four force plates (Infinity-T, BTS Bioengineering, Milan, Italy) at a sampling frequency of 400 Hz and off-line analyzed by means of custom-made software to obtain the GRF-derived indices. During gait assessments, the patients walked barefoot at a speed they considered comfortable for spontaneous walking and no further instructions were given to them.

As described in a previous study by Campanini and Merlo (Campanini and Merlo, 2009), DLA was computed as the mean value of the vertical component of the ground reaction force. It refers to the weight-bearing ability over the stance foot. DPA was computed as the mean value of the positive portion (forward direction) of the fore-aft component of the ground reaction force. DPA refers to the ability to get propulsion from the stance foot (Campanini and Merlo, 2009). Both DLA and DPA are expressed as percentages of body weight (%BW). At least three trials per patient were used in this study and the indices' median values were computed and used for further analyses.

### Statistical Analysis

First, descriptive statistics were used to analyze the data. Age, sex, clinical scores, and the values of instrumental indices were reported either by count or by the mean, standard deviation (SD), and range. Next, according to the aim of the study, a correlation analysis was carried out between DLA, DPA, and the structure- (RoMs, MTS) and activity-related (FAC, RMI, WHS, walking speed) variables. The non-parametric Spearman correlation index was used for all correlations because of the presence of ordinal variables and non-Gaussian distributed continuous variables. The statistical significance was set at 5%.

## Results

Data from 40 subjects were used in the study. There were 20 men and 20 women, with a mean age of 51 (12) years. The complete demographic and clinical data of the sample patients are reported in Table 1.

**Table 1:** Demographic and clinical characteristics of the sample patients.

**Characteristic**	***N*** **= 40**
Age	51 (12); 22–77
Sex, M/F	20/20
Affected side, Center/Right	20/20
Stroke onset, years	5.6 (7.0); 0.7–34.5
pADF Knee_0, deg	−6 (11); −40–10
pADF Knee_90, deg	+6 (11); −40–20
aADF Knee_0, deg	−25 (14); −55–5
aADF Knee_90, deg	−8 (12); −40–15
MTS PF Knee_0	3; 0–4
MTS PF Knee_90	3; 0–4
FAC	4; 2 – 5
RMI	12; 8–15
WHS	5; 3–6
Used orthoses	20 None; 12 AFO; 2 Peromed; 1 Dictus; 1 Foot-up; 4 other/customized

*Data are reported as mean (std) and range for age, angles, and years from stroke onset, as median and range for clinical scale scores, and as count for sex, affected side, and used orthoses*.

In the sample, walking speed was on average 0.45 (0.20) m/s, which is approximately one-third of the normal reference, and ranged between 0.1 and 1.1 m/s, indicating a level of impairment ranging from very severe to very mild (Bowden et al., 2008). Once normalized, the walking speed was, on average, 27 (12) %height/s and ranged between 5 and 68 % height/s. Despite their slowness, the sample patients exhibited—on average—moderate walking ability when assessed by clinical scales (see Table 1). Actually, out of 40 patients, 34 walked independently on level ground (FAC ≥ 4), 24 were community walkers (WHS ≥ 5), and 22 were able to walk on grass and uneven surfaces (RMI ≥ 12). These characteristics are common for post-stroke walking patients who are referred to rehabilitation services, thus supporting the external validity of the following results.

**Figure 1:**
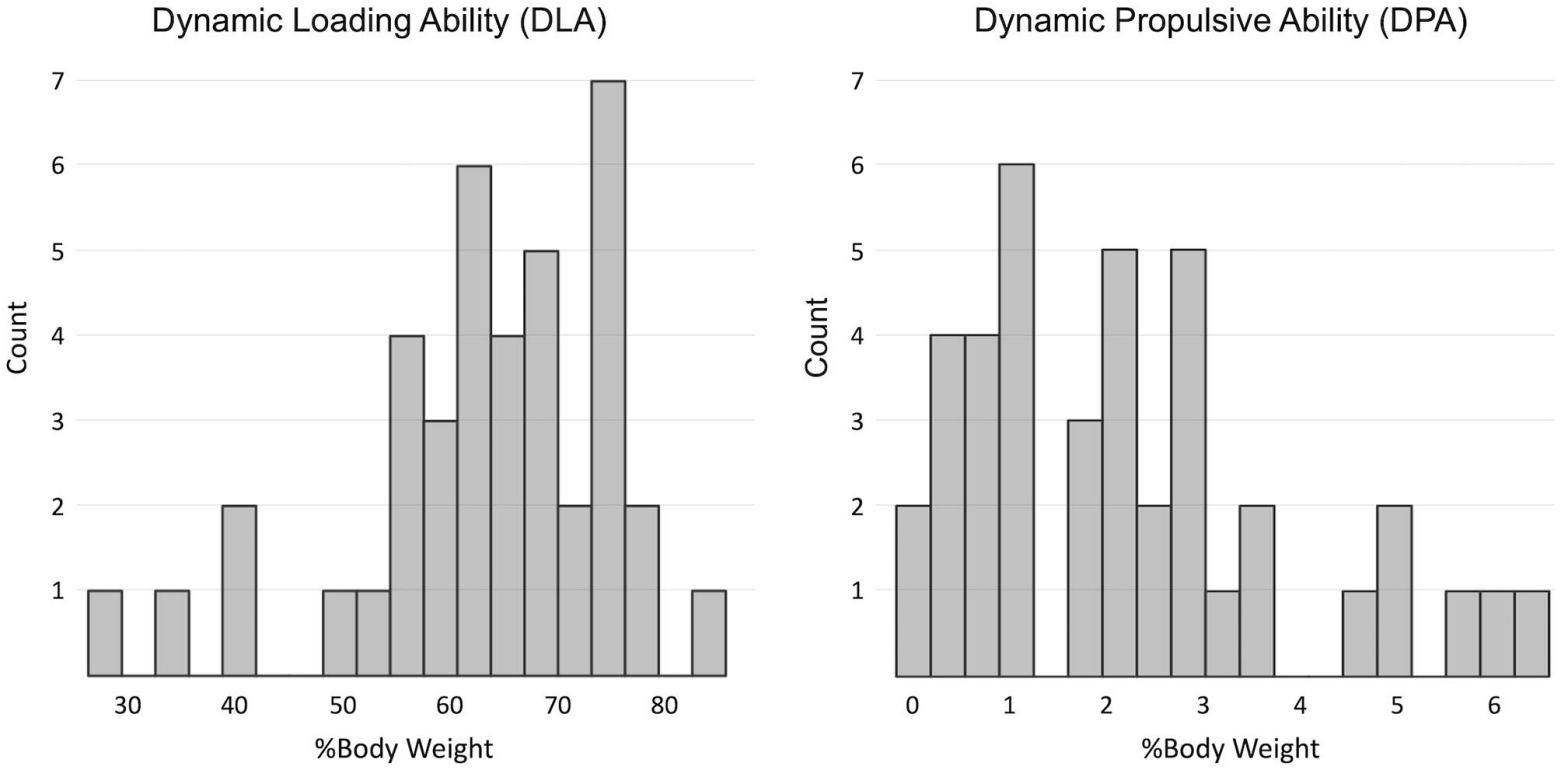
Distribution of indices of dynamic loading ability (DLA) and dynamic propulsive ability (DPA) in the sample.

The DLA was on average 63 (12) %BW and ranged between 27 and 83 %BW. The DPA was, on average, 2.2 (1.7) %BW and ranged between 0 and 6.4 %BW. The DPA and DLA distributions are shown in Figure 1.

**Figure 2:**
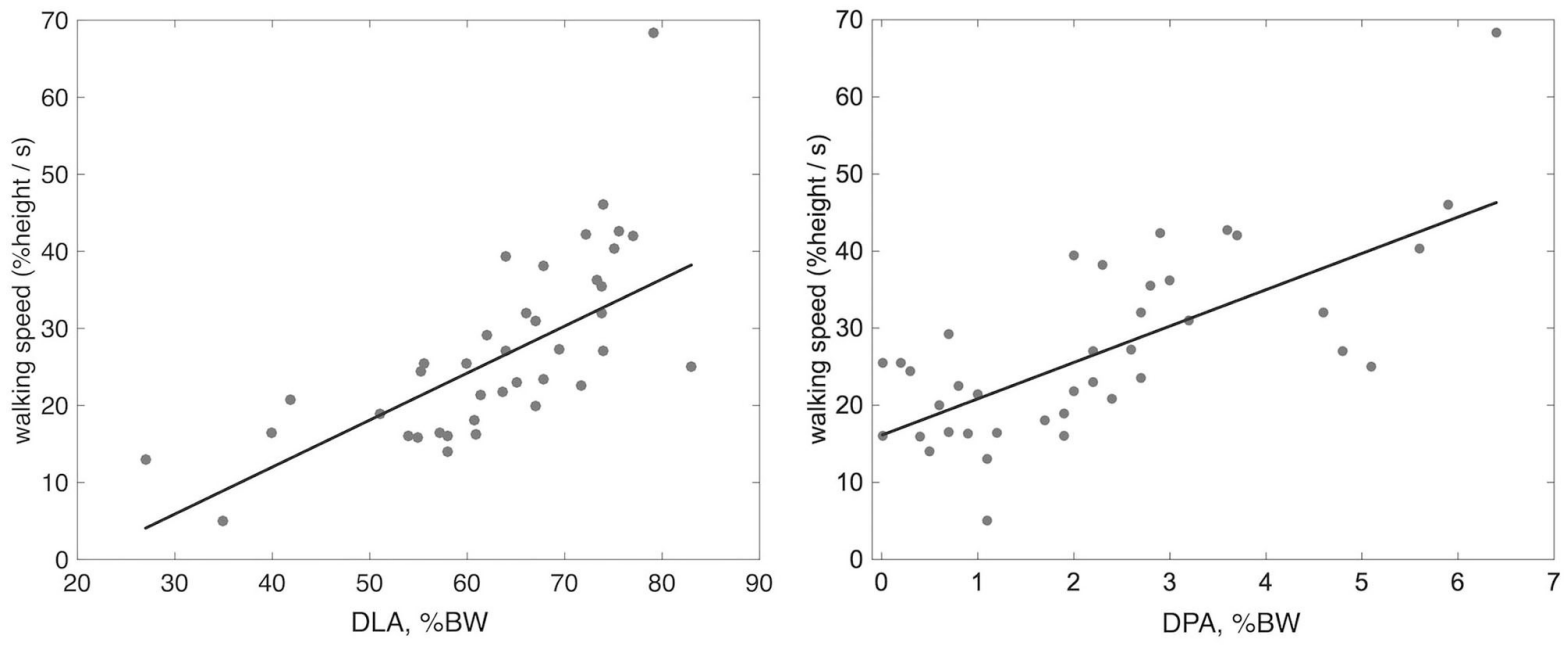
Scatter plot with robust regression line between height normalized walking speed and the indices of DLA and DPA.

The correlation between the investigated clinical and instrumental variables is reported in Table 2. The scatterplots between walking speed, normalized by height, and both DLA and DPA are presented in Figure 2, along with the line of robust regression and the linear relationship between the indices and gait velocity.

**Table 2:** Spearman's correlation between variables typically assessed during clinical evaluation of patients with chronic stroke, related to both *Body Structures and Function* and *Activity* domains of the International Classification of Functioning, Disability and Health, and GRF-based indices of dynamic loading ability (DLA) and dynamic propulsive ability (DPA) during walking at spontaneous speed.

	**Vel (%height/s)**	**pADF Knee_0, deg**	**pADF Knee_90, deg**	**aADF Knee_0, deg**	**aADF Knee_90, deg**	**MTS PF Knee_0, deg**	**MTS PF Knee_90, deg**	**FAC**	**RMI**	**WHS**
DLA, %BW	0.796^***^	0.131	0.389^*^	0.458^**^	0.380^*^	−0.436^**^	−0.480^**^	0.296	0.328^*^	0.361^*^
DPA; %BW	0.696^***^	0.239	0.443^**^	0.460^**^	0.380^*^	−0.272	−0.478^**^	0.473^**^	0.440^**^	0.474^**^
FAC	0.275	0.221	0.274	0.342^*^	0.359^*^	0.003	−0.229	—		
RMI	0.515^***^	0.324^*^	0.356^*^	0.332^*^	0.308	−0.037	−0.189	0.770^***^	—	
WHS	0.395^*^	0.163	0.255	0.329^*^	0.286	0.050	−0.109	0.832^***^	0.838^***^	—

* *p < 0.05*,

** *p < 0.01*,

*** *p < 0.001*.

*Vel: height normalized walking velocity; pADF Knee_0: maximum passive Ankle DorsiFlexion, with the knee extended; pADF Knee_90: maximum passive Ankle DorsiFlexion, with the knee flexed; aADF Knee_0: maximum active Ankle DorsiFlexion, with the knee extended; aADF Knee_90: maximum active Ankle DorsiFlexion, with the knee flexed; MTS PF Knee_0: Modified Tardieu Scale at the Plantar Flexor muscles, with the knee extended; MTS PF Knee_90: Modified Tardieu Scala at the Plantar Flexor muscles, with the knee flexed; FAC: Functional Ambulatory Categories; RMI: Rivermead Mobility Index; WHS: Walking handicap Scale; %BW: percentage of the Body Weight*.

## Discussion

In this study, we examined the relationship between the instrumental indices of loading and propulsive ability during gait and a set of clinical assessments associated with walking ability in a sample of patients with chronic stroke. The main finding is that DLA and DPA are correlated with all clinical variables; the only exception being passive ankle dorsiflexion measured with the extended knee. In most cases, correlations were significant at the 0.01 level.

Among all investigated variables, the strongest correlation with DLA and DPA was found when measuring walking velocity, with *rho* values similar to those reported by Campanini and Merlo (Campanini and Merlo, 2009). An increase in propulsion resulted in increased walking velocity (rho = 0.7, *p* < 0.01). The increase in velocity resulted in an increase in both the rising front and the peaks of the vertical GRF, which are detected by DLA (rho = 0.8, *p* < 0.01).

The correlation between indices and both ankle RoMs and ankle spasticity was in the order of 0.4 (see Table 2). This indicates a moderate association between the impairment assessed at the bedside and the weight-bearing and propulsive ability of the patient. No relationship was found between the assessment of triceps surae spasticity at the bedside and any of the scales assessing either activity or participation. As pointed out in recent literature, this confirms that the amount of triceps spasticity assessed at the bedside is not the primary cause of walking impairment in patients with acquired lower limb deformities including EVD, EVFD, and SKG (Gracies, 2005; Baude et al., 2019; Merlo and Campanini, 2019).

When focusing on clinical scales, the findings outline the difference in correlation strength among walking speed, FAC, RMI, and WHS, as can be seen both in Table 2 and in Figure 2. The covariation is especially evident for RMI, which focuses more on function rather than on the amount of assistance (FAC) and the type of surroundings needed for walking (WHS). Propulsion of the paretic limb is key in the daily life of the patients, as can be observed in Table 2, where DPA proved to be correlated with FAC, RMI, and WHS at the 0.01 level with *rho* approaching 0.5. Moreover, previous investigations have shown that changes in paretic propulsion correlate with the amount of lower limb muscle activity (Turns et al., 2007), walking speed (Raja et al., 2012) and walking endurance when assessed with the 6 min walking test (Awad et al., 2015).

In patients with stroke, the impairment at the gastro-soleus complex due to muscle shortening increases muscle stiffness and, in some cases, muscle overactivity affects both foot placement and balance during the stance phase, thus limiting the ability to bear the weight over the stance foot, as measured by DLA. Conversely, an extensible triceps surae allows for better foot support during stance and the forward rotation of the tibia over the stance foot (i.e., the second rocker). This results in a forward tilted GRF, which in turn generates forward propulsion, as assessed by DPA (Campanini et al., 2013). DLA and DPA encompass many different features related to body structures and body functions (e.g., ankle RoMs) that result in weight-bearing and propulsive abilities, which are essential to walking efficiency and speed.

### Use of DLA and DPA in Clinical Routine Assessments

The value of GRF-based indices lies in the extreme ease of this instrumental examination and in the ability to also evaluate patients who wear orthoses or use walking aids. Normative values for DPA and DLA are available in the literature and can be used when assessing patients (Campanini and Merlo, 2009).

The ability of the paretic limb to generate propulsion during walking is a critical determinant of long-distance walking function after a stroke (Awad et al., 2015).

During the rehabilitation period, DLA and DPA can be used to test whether the increased speed of the gait of the patient is due to a real recovery in the propulsion of the affected and treated limb, or due to an increase in compensation of the contralateral limb (Morita and Yamamoto, 1995). Particularly, in hemiparetic patients, the same increase in walking speed can be obtained either through the recovery of the impaired limb—as targeted by the rehabilitative intervention—or through compensatory mechanisms with greater propulsion from the contralateral limb. These two opposite behaviors can be easily recognized by the GRF-based indices, thus providing valuable feedback on the effectiveness (or lack thereof) of the ongoing rehabilitation. Although an increase in walking speed is a positive result, regardless of how it is achieved, quantitative feedback on the treatment effectiveness in terms of propulsion would allow practitioners to corroborate or modify the ongoing rehabilitative treatment. GRF-based indices further provide clinical cues, such as highlighting the presence of a braking mechanism of the lower limbs. When walking speed remains limited despite adequate propulsion, a brake must exist. This typically occurs at the knee, where flexion is stopped by the reflex activation of the quadriceps prompted precisely by the rapid knee flexion consequent to propulsion (Campanini et al., 2013; Merlo and Campanini, 2019).

### Study Limitations

Univariate analysis of variable correlations was conducted for this study in line with the limited sample size (*n* = 40). However, to estimate the extent of these correlations, when the set of variables analyzed is mutually related, a multivariate approach would be more appropriate. Future studies based on larger samples could address this topic.

### Conclusions

Our results support the use of DLA and DPA when measuring the overall walking ability in patients with chronic stroke. Thanks to the ease in obtaining these indices, their validity, and the availability in the literature of both their normal values and minimal detectable change, professionals could use them to quantify and monitor the motor recovery of patients with stroke during their rehabilitation.

## Data Availability Statement

The raw data supporting the conclusions of this article will be made available by the authors, without undue reservation.

## Ethics Statement

This study was reviewed and approved by CEROM, protocol 5953/2017. The patients provided their written informed consent to participate in this study.

## Author Contributions

DM and AM: conceptualization, methodology, and supervision. GB, PP, MG, FM, CR, and PZ: data curation. AM, DM, and GB: formal analysis and writing–original draft. AM, DM, IC, and GB: writing–review and editing. All authors contributed to the article and approved the submitted version.

## Conflict of Interest

The authors declare that the research was conducted in the absence of any commercial or financial relationships that could be construed as a potential conflict of interest.

## Publisher's Note

All claims expressed in this article are solely those of the authors and do not necessarily represent those of their affiliated organizations, or those of the publisher, the editors and the reviewers. Any product that may be evaluated in this article, or claim that may be made by its manufacturer, is not guaranteed or endorsed by the publisher.
